# Vascular cognitive impairment (VCI): Progress towards knowledge and treatment

**DOI:** 10.1590/S1980-57642010DN40100002

**Published:** 2010

**Authors:** Silvia Di Legge, Vladimir Hachinski

**Affiliations:** 1 MD, PhD, Stroke Unit, Department of Neuroscience, University Tor Vergata, Rome, Italy; 2 MD, FRCPC, DSc, Department of Clinical Neurological Sciences, London Health Sciences Centre (LHSC) and University of Western Ontario (UWO), London, ON, Canada.

**Keywords:** cerebrovascular disease, dementia, vascular cognitive impairment, diagnostic criteria, doença cerebrovascular, demência, comprometimento cognitivo vascular, critérios diagnósticos

## Abstract

Until recently, the study of cognitive impairment as a manifestation of cerebrovascular disease (CVD) has been hampered by the lack of common standards for assessment. The term vascular cognitive impairment (VCI) encompasses all levels of cognitive decline associated with CVD from mild deficits in one or more cognitive domains to crude dementia syndrome. VCI incorporates the complex interactions among classic vascular risk factors (i.e. arterial hypertension, high cholesterol, and diabetes), CVD subtypes, and Alzheimer’s Disease (AD) pathology. VCI may be the earliest, commonest, and subtlest manifestation of CVD and can be regarded as a highly *prevalent and preventable syndrome*. However, cognition is not a standardized outcome measure in clinical trials assessing functional ability after stroke. Furthermore, with the exception of anti-hypertensive medications, the impact of either preventive or acute stroke treatments on cognitive outcome is not known. Although clinical, epidemiological, neuroimaging, and experimental data support the VCI concept, there is a lack of integrated knowledge on the role played by the most relevant pathophysiological mechanisms involved in several neurological conditions including stroke and cognitive impairment such as excitotoxicity, apoptosis, mitochondrial DNA damage, oxidative stress, disturbed neurotransmitter release, and inflammation. For this reason, in 2006 the National Institute of Neurological Disorders and Stroke (NINDS) and the Canadian Stroke Network (CSN) defined a set of data elements to be collected in future studies aimed at defining VCI etiology, clinical manifestations, predictive factors, and treatment. These recommendations represent the first step toward developing diagnostic criteria for VCI based on sound knowledge rather than on hypotheses. The second step will be to integrate all studies using the agreed methodologies. This is likely to accelerate the search for answers.

## Abstract (pt)

Até recentemente, o estudo do comprometimento cognitivo como manifestação de doença cerebrovascular (DCV) tem sido comprometido pela falta de métodos de abordagem. O termo comprometimento cognitivo vascular (CCV) inclui todos os níveis de declínio cognitivo associados à DCV, de déficites discretos em um ou mais domínios cognitivos a uma síndrome demencial inequívoca. CCV incorpora interações complexas entre fatores de risco vasculares clássicos (i.e. hipertensão arterial, colesterol elevado e diabetes), subtipos de DCV e patologia de Doença de Alzheimer (DA). CCV pode ser a mais precoce, comum e sutil manifestação de DCV e pode ser encarada como uma demência altamente prevalente e prevenível. Todavia, a cognição não é uma medida de desfecho em ensaios clínicos que avaliam habilidades funcionais após acidente vascular cerebral (AVC). Além disso, com exceção de medicações anti-hipertensivas, o impacto tanto de medidas preventivas quanto tratamento agudo de AVC em desfecho cognitivo não é conhecido. A despeito de que dados clínicos, epidemiológicos, de neuroimagem e experimentais suportam o conceito de CCV, há falta de conhecimento integrado no papel desempenhado pelos mecanismos patofisiológicos mais relevantes envolvidos em muitas condições neurológicas incluindo AVC e comprometimento cognitivo, como excitotoxicidade, apoptose, dano ao DNA mitocondrial, stress oxidativo, distúrbios na liberação de neurotransmissores e inflamação. Por esta razão, em 2006 o *National Institute of Neurological Disorders and Stroke (NINDS) and the Canadian Stroke Network (CSN)* definiram um conjunto de dados a serem coletados em estudos futures auxiliando na definição da etiologia, manifestações clínicas, fatores preditivos e tratamento. Estas recomendações representam o primeiro degrau para o desenvolvimento dos critérios diagnósticos para CCV baseados em conhecimentos mais do que em hipóteses. O segundo degrau seria integrar todos os estudos usando metodologias aceitas, o que aceleraria a busca por respostas.

## Recommendations for developing common standards for the study of VCI: summary of the 2006 NINDS/CSN workshop

Cerebrovascular disease (CVD) and cognitive impairment are strictly associated. If we approach them at their extremes, i.e. clinical stroke and overt dementia, we know that stroke, dementia, or both diseases affect one in three persons in North America.^1^ Dementia occurs in up to one third of patients with a previous stroke,^2-4^ while some degree of cognitive impairment may be detected in up to two-third of stroke patients.^5^ Growing evidence suggests that cognitive impairment related to CVD (i.e. silent or overt stroke and vascular risk factors) and Alzheimer’s disease (AD) share common elements:

i) cognitive impairment of the AD-type may be pre-existing to stroke diagnosis in about one sixth of patients;^6^
ii) CVD and AD pathology coexist in most of dementia cases;^7-10^
iii) prevalent or incident CVD or vascular risk factors may trigger or exacerbate a dementia of the AD-type;^11-13^ and
iv) cerebral amyloid angiopathy (CAA) impairs blood vessel function and induces morphological changes in humans and animals.^14^

The term vascular cognitive impairment (VCI)^15-17^ refers to any cognitive impairment caused by or associated with vascular risk factors, and encompasses a cognitive spectrum ranging from mild cognitive impairment to overt dementia. The VCI concept also embodies the link between CVD and AD, and carries the potential for prevention. However, current knowledge on VCI is still insufficient to develop definite diagnostic criteria.

With the aim of developing common standards, which represents the first step towards definition of evidence-based diagnostic criteria for VCI, in 2006 the National Institute of Neurological Disorders and Stroke (NINDS) and the Canadian Stroke Network (CSN) defined a minimum and common set of data elements to be collected in future clinical and research studies.^18^ Recommendations were developed by working groups for clinical diagnosis, epidemiology, neuropsychology, brain imaging, neuropathology, experimental models, biomarkers, genetics, and clinical trials. Briefly, these recommendations comprise:

### Clinical/epidemiology section

A working group approached the issue of VCI from two perspectives. First, the group focused on the items that should be collected in an ideal population-based study on VCI; second, researchers developed recommendations for defining a subgroup of variables to be practically oriented for the clinician. The two sets of items include demographics, informant (as additional source of information), family history, health history, and evaluation (including the subjective impression and symptoms of the evaluated subject, a physical examination, and laboratory data).

### Neuropsychology section

The discussion was centered on developing test protocols to be used in multi-center trials and aimed at detecting cognitive changes that are peculiar of VCI (i.e. executive dysfunction, such as slowed information processing, impairment in the ability to shift from one task to another, and impairment in the ability to hold and manipulate information). Three separate test protocols were proposed: a 5-minute test battery to be used for quick screening; a 30- and a 60-minute test battery to better characterize the number and severity of domains involved. These protocols were offered as a basic assessment, appropriate for different purposes, and need validation.

### Neuroimaging

There are no imaging criteria for VCI diagnosis. So far, only descriptions of brain changes found to be associated with VCI have been described. A common and minimal set of data to be shared by all investigators was recommended to allow comparisons between the studies and facilitate the pooling of data. MRI (minimum required: 1.0 T) represents the ideal tool for studying cognitive disorders because of its sensitivity and the great amount of data rendered by a single study. An informative MRI study should contain at least: 3D T1-weighted, T2-weighted, fluid-attenuated inversion recovery (anatomy and presence of infarcts or other pathology), gradient-echo (large or small, acute or chronic haemorrhages), and diffusion-weighted images (acute stroke and integrity of white matter fibers). Criteria that differentiate perivascular space and infarcts were based on the Cardiovascular Health Study (CHS).^19^ Criteria for CT-based studies have also been defined. Areas for future imaging research based on other neuroimaging techniques and applications have been discussed.

### Neuropathology

References for optimal brain handling and processing at autopsy to encourage uniformity of autopsy protocols across centers were provided: the Alzheimer’s Disease Research Centers, USA (ADRC) guidelines adapted from those used by many neuropathologists participating in the Consortium to Establish a Registry for Alzheimer’s Disease (CERAD) protocol for sampling of fixed brain,^20^ and the National Alzheimer’s Coordinating Center (NACC; www.alz.washington.edu) for guidance in the assessment of CVD (i.e. parenchymal lesions, and vascular abnormalities that may have caused them). Vascular risk factors causing brain lesions in the absence of severe CVD should be added, as well as AD and hippocampal lesions, which are not obviously associated to CVD or vascular risk factors. Guidelines have been proposed for a minimal informative dataset and an ideal dataset for each type of abnormality to be considered: atherosclerosis of the basal, peripheral and meningeal vessels, microvascular disease (i.e. arteriosclerosis, cerebral amyloid angiopathy, miscellaneous microangiopathies), parenchymal abnormalities associated with CVD, leukoencephalopathy, hippocampal lesions, incomplete ischemic injury, mixed vascular and parenchymal pathology.

### Experimental models

Animal models of VCI are necessary to better understand CVD mechanisms contributing to impaired brain function and cognition. Animal models can help understanding how molecules, cells, and systemic conditions (i.e. hypertension, diabetes) participate in vascular and parenchymal injury. The use of these models is expected to help discover biomarkers and disease mechanism-linked targets for diagnostic and therapeutic purposes, thus facilitating early identification and intervention in VCI. Several experimental models are available to study VCI. However, as a consequence of lack of standardized diagnostic criteria, these models are not as well developed as those adopted for studying acute ischemic injury and AD. Available experimental models in rodents and primates would provide further insight into the mechanisms that produce VCI including: β-amyloid deposition (Aβ) and cerebral amyloid angiopathy (CAA), vascular pathology and vascular reactivity changes of cerebral autosomal arteriopathy with subcortical infarcts and leukoencephalopathy (CADASIL), cerebrovascular insufficiency (i.e. evolution and consequences of chronic ischemia in bilateral carotid ligation, and hypertensive vasculopathy in spontaneously hypertensive, stroke-prone animals), models of the normal aging brain, and cellular mechanisms underlying white matter changes through ex-vivo slices of different white matter regions of the brain. Further, animal models may allow definition of the cognitive and behavioural changes associated to pathological findings.

### Biomarkers

The diagnosis of VCI could be facilitated by the availability of reliable biomarkers that can be measured in body fluids such as blood and cerebrospinal fluid (CSF). This would allow improved separation between VCI and AD cases. However, this ideal endeavour is hampered by the heterogeneous phenotype and etiology of VCI and by the concomitant presence of CVD pathology in the brains of AD patients. Moreover, searching for biomarkers is hampered by the huge variability between individuals. Candidate CSF markers for VCI include: the serum albumin ratio, to identify blood-brain barrier damage to the small intravascular vessels; sulfatide, to identify demyelination of white matter; neurofilament, to identify axonal degeneration (marker of white matter damage); and matrix metalloproteinases (MMPs), to identify changes in the extracellular matrix associated with CVD (i.e. vascular disease with inflammation). All these markers have been found to be elevated in patients with subcortical ischemic vascular dementia (SIVD). Most of these markers have been found to be high in patients with VCI but not in those with AD. Conversely, elevated levels of tau and phosphor-tau proteins are not found in VCI patients, yet can identity patients with AD. All these tests need to be validated in VCI patients.

### Genetics

Genetic determinants of VCI may include genes implicated in hypertension and carotid artery disease (both associated with stroke and cognitive impairment), and genes that affect the response of the brain to vascular disease (i.e. vascular tolerance and neuronal plasticity). Monogenic disorders with known genetic factors which are associated with VCI-i.e. CADASIL, hereditary variants of CAA, sickle-cell disease, Fabry disease, and homocystinuria- may be useful because they provide homogeneous cohorts for a certain vascular pathology. Other presumed inherited conditions that could be potentially useful for VCI study are cerebral autosomal recessive arteriopathy with subcortical infarcts and leukoencephalopathy (CARASIL), cerebroretinal vasculopathy, and hereditary endotheliopathy with retinopathy, nephropathy, and stroke (HERNS). In some of these diseases the genes have been mapped to specific chromosomal regions, but the underlying genetic defect has not yet been identified. Genetic risk factors for VCI may be identified by link analysis or association studies. Limitations of genetic studies are: complexity of the phenotype of VCI (definition of target phenotypes and protocols used for phenotypic assessment need to be defined and validated), selection of appropriate controls (requiring population stratification for several variables, including ethnicity), selection of candidate genes/candidate gene regions, and replication in independent cohorts of patients and controls. Although technically feasible, genetic association studies are not realistic, at least currently. In fact, due to the complexity of the VCI phenotype, these studies would be very expensive, because they require a large sample size (i.e. several thousands of individuals) and the collaborative effort of multiple centres.

### Clinical trials

Post-stroke cognitive impairment and dementia have been linked to poor long-term survival.^21^ Additionally, cognitive impairment after stroke reduces quality of life and predicts institutionalization better than age or physical impairment. Cognitive deficits may be severe when other impairments are only mild or moderate. Despite this evidence, primary outcomes after stroke generally include recurrent stroke, myocardial infarction, or vascular death, and measures of motor and functional ability to carry out the usual activities of daily living prevail over evaluation of cognitive functions. It follows that cognition should be an outcome measure in clinical trials on CVD and stroke. If the research community could agree to adopt the proposed 5-, 30-, and 60-minute cognitive test batteries in their protocols as the minimum acceptable standard, there would be a common basis for study-comparison and knowledge advancement. Tests for cognitive functions have three uses in clinical trials:

1) for testing subjects to determine eligibility for trials;
2) as a primary or secondary outcome measure, and
3) for determining adverse effects.

Since recommendations on VCI have been developed and made available for discussion in 2006, further steps in the whole field of cognitive impairment and dementia have been achieved and expert reviews focusing on different aspects of the complex pathophysiological mechanisms involved in VCI have been published. The present review outlines new information on this subject as well as predictions regarding future research directions allowed by ongoing improvements in knowledge and technology.

## Advances in the study of VCI: progress update

### Imaging studies in VCI

VCI is caused by different stroke subtypes, including large vessel disease with strategic single and multiple strokes and small vessel disease with progressive damage to deep white matter.

The relevance of location and number of infarcts with respect to cognitive function has been recently addressed in two studies. In the first study, this association was investigated in the Baltimore Longitudinal Aging Study Autopsy Program, a prospective study on the effects of aging on cognition and dementia.^22^ Among 122 men and 57 women (92% white, 17.5 years of education, and a mean age at death of 86.9 years), the odds of dementia of the AD type were increased by both asymptomatic and symptomatic hemispheral infarcts. In this study, the authors observed that:

i) the number but not size of macroscopic hemispheral infarcts increased the odds of dementia;
ii) macroscopic and microscopic infarcts contributed equally to the risk of developing dementia; and
iii) hemispheral infarcts, whether silent or clinically manifest, alone or in conjunction with AD pathology, accounted for 35% of dementia cases.

These findings support a synergism between AD and vascular pathology and the importance of burden and location of infarcts. The study also showed that dementia was not increased by deep subcortical infarcts or risk factors for stroke (such as coronary artery disease or severe hypertension) in the absence of infarcts. In the second study,^23^ the authors observed that among 4,030 non-demented participants of the Age Gene/Environment Susceptibility-Reykjavik Study (AGES-Reykjavik), having infarcts in more than 1 location was found to be associated with poor performance in memory, processing speed, and executive function, independent of cardiovascular comorbidities, white matter lesions, and brain atrophy. This study suggested that the number and distribution of infarcts jointly contribute to cognitive impairment.

Chronic subcortical vascular disease is epidemic in the aging population and is associated with VCI. The cognitive phenotype of this chronic small vessel disease includes slowed information processing, impaired executive function, and gait difficulty. Typical MRI findings are periventricular (PV) white matter changes seen as hyperintensities (WMHs) on T2-weighted images, which represent extensive regions of demyelination, inflammatory cells around damaged blood vessels, and lacunar infarcts.

In a recent study, volumetric progression of WMH on MRI was found to be a predictor of cognitive impairment^24^. In particular, the authors observed that every 1-mL/year increase in PV WMH volume increased the risk of cognitive impairment by 94%. This analysis was performed in 98 community-dwelling individuals aged 65 years or older who were functionally independent and not cognitively impaired when included in the longitudinal Oregon Brain Aging Study. Demographics, vascular risk factors (hypertension, diabetes, stroke, or TIA), and APOE 4 status were collected. A score > 0.5 on the Clinical Dementia Rating Scale (CDRS) was the criteria adopted to define cognitive impairment. Image analysis included quantitative assessment of regional brain volumes of interest: PV WMH, subcortical WMH, total WMH (PV plus subcortical WMH), brain volume, ventricular cerebral spinal fluid, and hippocampal volumes. MRI volume analyses were adjusted for baseline hippocampal volumes. Over a 10-year follow-up, 48% of patients developed at least one vascular risk factor (32% hypertension), and 53/98 (54%) developed cognitive impairment with no dementia. AD pathology was the most common diagnosis, while CVD was seen in almost all cases. In this study the authors introduced the term “persistent cognitive impairment” to indicate mild cognitive impairment with no dementia and adopted the CDRS criteria for cognitive evaluation, which are biased towards AD diagnosis, memory being the primary category for defining severity.

Currently, no single clinical feature or diagnostic test is sufficient to identify patients with the small vessel form of VCI. WMHs on T2-weighted MRI are thought to be caused by ischemic insults related to hypoperfusion of the vulnerable deep white matter secondary to hypertension, diabetes, and other vessel diseases. However, growing evidence suggest disruption of the blood-brain barrier secondary to inflammatory response. Innovative neuroimaging techniques, such as Diffusion Tensor Imaging (DTI), MR Spectroscopy (MRS), functional MRI, and β-amyloid PET, can investigate microstructural integrity, molecular biology, and activation patterns, providing new insights into the relationships between brain and cognition.^25^ In particular, MRS allows investigators: to discriminate among dementias through different patterns of brain metabolic changes; to follow-up changes in longitudinal studies; to correlate changes of brain metabolic patterns with changes in clinical markers of disease progression; and to evaluate the effects of treatments through brain metabolic changes.^26^ In humans, the development of β-amyloid binding PET ligands offers, for the first time, the ability to measure parenchymal and vascular β-amyloid deposition using a serial longitudinal approach. With improved in vivo measurements of β-amyloid, physiological investigations of vascular reactivity, and longitudinal study designs, it should be possible to more firmly establish the role of blood vessel function in producing impairment in CAA and AD. Identification of vascular dysfunction as a significant component of CAA and AD may pave the way for new therapeutic approaches for this hitherto largely untreatable disease.^14^ A recent study with contrast-enhanced MRI has detected a group of patients with VCI who have increased permeability to gadolinium DTPA in the white matter compared with age-matched control subjects.^27^ These preliminary data support the concept that a subset of patients with VCI could have an inflammatory process with disruption of the blood-brain barrier (BBB).

## Risk factors for VCI

### Vascular risk factors

The impact of the presence and number of vascular risk factors (VRF) on cognitive function has been recently investigated in a cross-sectional analysis of the Canadian Study of Health and Aging (CSHA) baseline data.^28^ In this analysis, 577 non-demented elderly individuals aged 65 years old or older were divided into 3 groups: a reference group (0 VRF; n=82); intermediate brain-at-risk group (1-2 VRF; n=360); and a high brain-at-risk group (=3 VRF; n=135). Elderly individuals presenting with =3 VRF showed greater impairment on measures of executive functions/processing speed than participants with no VRF.

The German SEARCH study^29^ investigated the impact of hypertension on cognitive performance in 525 non-demented community-dwelling individuals (mean age 65 y.o., range 40-85). Individual vascular risk was based on glycosylated haemoglobin, serum cholesterol, high sensitive C-reactive protein, body mass index, smoking pack/years, and blood pressure. The authors observed that midlife systolic blood pressure explained up to 11% of the variance in cognitive performance. These findings suggest that in non-demented community-dwelling individuals, hypertension may account for one tenth of cognitive impairment and thus for an increased risk for dementia.

There is a growing evidence of a link between diabetes, vascular damage, and neurodegenerative changes in the brain. Type 2 diabetes interacts with aging, hypertension, Ab deposition, and the APOE 4 allele. Three population-based studies have recently addressed this issue. In the first of the studies, diabetes increased the risk of dementia especially if the onset was in midlife.^30^ Subjects listed in the Swedish Twin Registry were enrolled in the HARMONY dementia study in 1998 when they at 65 years of age or older. A total of 467 participants were diagnosed with dementia: 292 with AD, 105 with vascular dementia (VaD), and 70 with other dementias. Diabetes was associated with a moderately increased risk for dementia, with a higher risk for VaD (OR, 2.17; 95% CI, 1.36-3.47). However, the onset of diabetes before the age of 65 years was associated with a 125% increased risk for AD. A limitation of this study was that the authors focused on dementia and no information could be provided on the risk of developing milder degrees of cognitive impairment. In the population-based Atherosclerosis Risk in Community (ARIC) study,^31^ the association between cardiovascular risk factors measured in midlife and risk of developing dementia was investigated. In 1990-1992, 11,151 participants aged 47-70 y.o. (23% African-Americans) underwent physical and neuropsychological examination. Over a twelve-year period, 203 participants had been hospitalized for dementia. Smoking (HR 95% CI 1.7), hypertension (HR 95% CI 1.6), and diabetes (HR )% CI 2.2) were strongly associated with increased risk of dementia, especially when risk factors were measured in individuals younger than 55 y.o., with no differences in ethnicity, thus extending the results of previous observational studies conducted in Caucasian and Asian populations to apply to African-Americans. In the Age Gene/Environment Susceptibility Reykjavik Study^32^ the authors explored, in 1,917 non-demented individuals (mean age 76 y.o.), which cognitive abilities were affected in type 2 diabetes individuals and whether undiagnosed diabetes and impaired fasting glucose were related to cognitive impairment. In this study, individuals with type 2 diabetes had poorer cognitive performance than normoglycemics, particularly in processing speed. Notably, individuals with undiagnosed diabetes had the lowest cognitive performances. All these studies confirm the previous findings in the Honolulu Asia Aging Study (HAAS)^33^ that provided evidence of the association of type 2 diabetes to impaired cognition and brain vascular and degenerative pathology (infarcts and hippocampal atrophy).

The association between high cholesterol levels and impaired cognition has been previously investigated in population-based longitudinal studies albeit with contradictory results. Recently, elevated cholesterol levels in midlife have been strongly associated with risk of developing AD and VaD three decades later. In this large population-based multiethnic cohort study,^34^ 9,844 individuals underwent detailed health evaluations from 1964 to 1973, when they were 40 to 45 years old, and dementia was ascertained both in 1994 and in 2007. AD diagnosis was detected in 469 participants, while 127 had VaD. After adjusting for age, education, race/ethnic group, sex, midlife diabetes, hypertension, body mass index, and late-life stroke, AD hazard ratios (HR) were 1.23 for midlife borderline cholesterol (200-239 mg/dL) and 1.57 for high cholesterol (≥240 mg/dL). VaD HR was 1.50 for borderline cholesterol and 1.26 for high cholesterol. This study further confirms that a vascular risk factor (i.e. hypercholesterolemia) is linked not only to VaD, but also to AD, suggesting a clinical, pathological, and vascular risk factor overlapping between the two conditions. However, in this study, dementia was obtained electronically from chart diagnosis based on the ICD-9 disease classification, which is biased towards AD, and pathological data were not available.

### Genetic risk factors

CADASIL is a genetically-determined pure form of subcortical ischemic vascular dementia (SIVD) with early onset. The condition has been invaluable in defining the profile and neuroimaging correlates of cognitive deficits in pure SIVD and in studying VCI, because it is often unaccompanied by the vascular risk factors typical of sporadic disease. In a 7-year follow-up study, an increase in lacunar infarcts, microbleeds, and ventricular volume, but not white matter lesions or atrophy, have been associated with cognitive decline -especially executive dysfunction- in younger-aged, mildly affected patients with CADASIL.^35^ Recently, a multicenter randomized trial on donepezil in CADASIL patients with VCI has been conducted.^36^ Although the study failed in addressing the primary end points, treated patients performed better on some executive tasks and timed measures, showing the feasibility of targeted therapeutic trials in narrowly defined subtypes of patients with VCI.

Polymorphisms in the gene for apolipoprotein E (APOE) influence the risk of developing AD and the deposition of Aβ within the brain. The APOE epsilon^4^ (APOE 4) allele is more conducive to vascular than parenchymal accumulation of Aβ in AD.^37^ The contribution of (APOE 4) to cognitive impairment after stroke is not yet fully understood. Recent data have been provided by two large community-based studies. In the first study, which analyzed data from the Canadian Study of Health and Aging (CSHA),^38^ the joint presence of stroke and APOE 4 was associated with a greater risk of dementia compared to the absence of these two factors. In particular, the adjusted hazard ratios (HR) of incident dementia, compared to individuals with neither stroke nor APOE 4, were 1.33 (95% CI 0.73-2.43) for stroke alone, 2.06 (95% CI 1.42-2.99) for APOE 4 alone, and 2.57 (95% CI 1.11-5.94) for both stroke and APOE 4. The study revealed that the effect of stroke on dementia did not seem to be modified by the presence of the APOE 4 allele. In the second study, the relationships of APOE genotype, stroke, and vascular risk factors with cognitive change were investigated in the Atherosclerosis Risk in Communities (ARIC) Study.^39^. Participants were recruited while at middle age: vascular risk factors were assessed during the baseline visit in 1990-1992; four cognitive assessments were performed between 1990-1992 and 2004-2006; and incident stroke was recorded over the 14 years of follow-up. There were 1,130 participants (mean age 59 years old; 62% women; 52% African-American) with longitudinal data. Diabetes, hypertension, previous stroke, and APOE 4 genotype independently contributed to cognitive decline in late middle age and early elderly years. A drawback of these studies is their lack of common standards for determination of cognitive profile, neuroimaging studies, and pathological confirmation of CVD and AD changes.

### Prevention of VCI

By the time a patient is diagnosed with dementia, the opportunity for prevention has passed. Conversely, the VCI concept is centered on the fact that vascular risk factors are treatable, and this should lead to a reduction in the incidence of cognitive impairment due to vascular causes. Evidence from prospective epidemiological studies suggests that optimizing the control of vascular risk factors such as hypertension, high cholesterol, diabetes, smoking and heart disease may prevent dementia. However, this has been proven in randomized-controlled trials only for hypertension.^40-44^ The effects of antihypertensive treatment on cognitive function was evaluated in 326 non-demented community-dwelling participants over the age of 70 years in the Women’s Health and Aging Study II.^45^ The use of angiotensin-converting enzyme inhibitors (ACE-Is) or diuretics, was associated with reduced incidence of impairment of both global and domain-specific cognition in elderly women, thus helping delaying the progression towards dementia. In particular, the authors observed that the use of either ACE-Is or diuretics for more than 3 years was associated with reduced incidence of impairment on the Mini-Mental State Examination (MMSE), Trail Making Test-Part A and Part B (TMT, Parts A and B), Hopkins Verbal Learning Test-Immediate Recall (HVLT-I), and Hopkins Verbal Learning Test-Delayed Recall (HVLT-D). The presence of vascular disease did not influence these effects.

A strong association between hypertension and its treatment, and the risk of developing dementia, was also recently addressed in the Honolulu-Asia Aging Study which followed a cohort of Japanese American men for an average of 32 years, with systolic BP (SBP), diastolic BP (DBP), and cognitive status measured at 6 examinations. Over a 32-year period, those who developed dementia had a greater increase followed by a greater decrease, in SBP, compared with non-demented subjects. Both of these trends were modified by antihypertensive therapy. Of the 1,890 men who completed all 6 of the exams, 112 diagnosed with incident dementia at examination 6 were compared to the 1,778 survivors without dementia. From midlife to late life, men who went on to develop dementia had an additional age-adjusted increase in SBP of 0.26 mm Hg (95% CI: 0.01 to 0.51 mm Hg) per year compared with survivors without dementia. These associations were strongest for VaD and were reduced substantially in men who were previously taking antihypertensive medication. Similar changes in diastolic BP were observed, but only for VaD, and the outcomes were not modified by antihypertensive treatment.^46^

Despite the complexity of VCI, alternative approaches to prevent cognitive impairment have been attempted. In this regard, we briefly mention the potential efficacy of participating in leisure activities as a possible alternative and additional strategy to preserve cognitive function. In the Bronx aging study (47), 71 of the 401 participants who were free of dementia or VCI at study-entry developed VCI (69% had VCI with no dementia) over a 21-year follow-up period. The researchers observed that participation in cognitive but not physical leisure activities was associated with lower risk of any degree of VCI. Future clinical trials testing the efficacy of leisure activities in reducing the incidence or severity of VCI, as well as exploring the effects of these activities on vascular risk factors and life-style changes, would be valuable.

### Epidemiology

The problem of assessing VCI and dementia in different races and ethnicities was identified in the 2006 consensus conference on VCI, as a lack of cognitive test validation across different languages and cultures.^18^ Until recently, the prevalence of dementia in developing countries has been estimated to be lower than in developed regions.^48^ Differences in the level of exposure to environmental risk factors (low levels of cardiovascular risk factors), high mortality in early life, and genetic factors have been advocated as possible explanation. However, the total number of individuals aged 60 and over in Latin America (LA) is estimated to reach around 98 million by 2025 (49) and cognitive impairment and dementia are also becoming a major public health problem in developing countries. Despite this evidence, epidemiological information on dementia remains scarce in these regions compared to developed regions of the world. In order to assess the prevalence of dementia and its causes across LA countries, data from eight population-based cohort studies have recently been analyzed.^50^ Based on this collaborative study, the global prevalence of dementia in the elderly (≥65 years) was found to be 7.1% (95% CI: 6.8-7.4), mirroring the rates of developed countries. However, the prevalence of dementia in relatively young subjects (65-69 years) was higher in LA studies (1.2%; 95% CI: 0.8-1.5) compared to prevalence observed in developed countries. AD was the most common cause of dementia observed in this study. The rate of illiteracy among the elderly was 9.3% and the prevalence of dementia in illiterates was two times higher than in literates (15.7% vs 7.2%; p<0.0001), suggesting a strong association between low educational level and lower cognitive reserve, possibly causing earlier emergence of clinical signs of dementia in the LA elderly population. This analysis also underlines the obstacles encountered by the researchers due to differences in the design of studies, diagnostic criteria adopted across countries and studies, and lack of homogeneity in imaging technique and criteria adopted. Furthermore, despite the authors´ expertise in the diagnosis of dementia in low educated individuals, and the fact that informant-based questionnaires used in all these studies were either adjusted for education level or had been specifically designed for the studied population, diagnosing dementia among illiterate and low educated individuals remains a difficult task and could lead to overestimation of dementia.

### Perspectives

The 2006 workshop on VCI (18) had the great advantage of bringing together researchers with different areas and levels of expertise in the study of cognition. They convened to review evidence, reach consensus, and make recommendations on a wide variety of diagnostic and management strategies to improve the understanding of the complex relationships existing between brain damage and the mechanisms involved in cognitive impairment.

In particular, the novel concept of VCI paves the way for a new approach to the study of the effects of determinants of vascular dysfunction, age-related changes, and degenerative brain lesions on cognition. However, because the clinical and pathophysiological correlates of VCI are not yet fully understood, leading experts in this field have delineated different lines of research. One of these lines focuses on the mechanisms of aging, AD, and hypertension, which number among the major determinants of cognitive dysfunction due to their deleterious effects on the structure and function of cerebral blood vessels. More specifically, aging changes the structure and vasodilatory capacity of vessels, increasing susceptibility to ischemia; vascular β-amyloid deposition (CAA), a common pathology of aging and in persons with AD, disrupts the vascular endothelium and myocytes obliterating capillaries and impairing autoregulation; chronic hyper-tension induces remodeling and stenosis of the arteries, fibrinoid necrosis of the arterioles, and impairs cerebrovascular reactivity. All these vasculopathic effects of aging, hypertension, and Aβ *deposition in the vessel and the brain*, acting alone or in concert, lead to hypoperfusion, blood brain barrier dysfunction, compromised protein synthesis and synaptic plasticity, and eventually neuronal death with cognitive consequences. This vascular dysfunction with reduction in blood flow could be the mechanism by which CAA is associated with microinfarction and small cortical infarcts.^14^ In particular, impaired delivery of energy substrates and nutrients to the active brain impedes the clearance of potentially toxic metabolic products. Reactive oxygen species derived from the enzyme NADPH oxidase are key pathogenic effectors of cerebrovascular dysregulation. This might lead, in turn, to cognitive impairment via cellular dysfunction and cell death.^51^ The role played by cerebrovascular oxidative stress in VCI suggests promise for new therapeutic opportunities to counteract and, possibly, reverse the devastating effects of cerebrovascular dysfunction on the brain, and thus warrants further study.

The study of VCI should also include detection of potential biomarkers of the disease. In this regard, novel potential markers for the study of VCI are under investigation. Asymmetrical dimethylarginine (ADMA), an inhibitor of endothelial nitric oxide synthase, is a marker of endothelial dysfunction and may be a potentially useful new biomarker of subclinical vascular brain injury, which is an important correlate of VCI and risk of stroke. Elevated circulating ADMA concentrations have been associated with systemic and carotid atherosclerosis, an elevated risk of developing stroke, and WMHs on magnetic resonance imaging. In the Framingham Offspring Study,^52^ higher plasma ADMA values were associated with an increased prevalence of subcortical infarcts, after adjusting for traditional stroke risk factors. Recent evidence also suggests that the cell adhesion molecule P-selectin (SELP) genotypes, which moderate circulating levels of P-selectin, may contribute to the adverse vascular processes that seem to promote cognitive impairment in individuals with cardiovascular disease.^53^ In addition, the recent hypothesis that a subset of VCI patients could have an inflammatory process with disruption and increased permeability of the BBB should be further investigated in order to detect the clinical spectrum, associated risk factors, and potential biological markers in the CSF of this subgroup of VCI (27). In this evolving scenario, the role of brain/tissue/biobanks (BTB-banks) in unravelling dementia in the coming decennia will identify, collect, preserve and type RNA and DNA extracted from brain/tissue/body fluids in order to update the pathological hallmarks of dementing disorders. Once identified, biomarkers in dementia will be incorporated into clinical drug trials and will help elucidate the mechanisms of the different dementing conditions as well drug actions.^54^

All the present evidence suggest that population-based studies and clinical trials based on common standards and focusing on defining the clinical spectrum of VCI, its major determinant and predictors, will increase our understanding and treatment of VCI. Also, conflicting data between epidemiological and small autopsy series on imaging factors associated with risk of developing cognitive impairment and dementia suggest this should be an area of continued investigations. Based on the results of recent population-based studies exploring the association between classic vascular risk factors and cognition, potential areas of interest for future investigation include the pathophysiology of diabetes and the brain, genetic contribution of diabetes and the brain, and the clinical and functional consequences of diabetes.^55^ Cognitive impairment should be considered as an outcome measure in diabetes studies and should be included by the American Diabetes Association (ADA) as a complication of type 2 diabetes in future treatment guidelines. Testing the effects of aggressive management of traditional vascular risk factors as well as of novel treatments will allow confirmation of epidemiological, clinical, and pathogenetic findings.

Given the growing interest in the field of cognition in the aging brain, the novel approach to the study of VCI outlined by the 2006 consensus conference^18^ should encourage researchers to adopt common standards in their studies to develop evidence-based diagnostic criteria for VCI, and provide more specific and useful answers.
